# Expression Profile of miRNA from High, Middle, and Low Stress-Responding Sheep during Bacterial Endotoxin Challenge

**DOI:** 10.3390/ani13030508

**Published:** 2023-02-01

**Authors:** Umesh K. Shandilya, Ankita Sharma, Danielle Naylor, Angela Canovas, Bonnie Mallard, Niel A. Karrow

**Affiliations:** 1 Department of Animal Biosciences, University of Guelph, Guelph, ON N1G 2W1, Canada; 2 Department of Pathobiology, Ontario Veterinary College, Guelph, ON N1G 2W1, Canada

**Keywords:** stress responder, microRNA, biomarker, lipopolysaccharide

## Abstract

**Simple Summary:**

Exposure to microbial stressors has huge implications in terms of animal health and welfare. Bacterial lipopolysaccharide (LPS) is a potent endotoxin, commonly responsible for various livestock pathologies, which impose huge economic losses to the livestock industry. MiRNAs regulate major biological processes and have multiple biological functions in terms of stress responsiveness. However, research on the altered miRNA expression profiles in sheep is still inadequate, especially in terms of the stress response to LPS. We found that extreme phenotypes, high and low stress responding sheep, had different expression patterns of miRNA. Our study indicated that miRNAs play an indispensable role in the stress response and could be potential biomarkers of stress.

**Abstract:**

Animals respond to stress by activating a wide array of physiological and behavioral responses that are collectively referred to as the stress response. MicroRNAs (miRNAs) are small, noncoding RNAs that play key roles in the regulation of homeostasis. There are many reports demonstrating examples of stress-induced miRNA expression profiles. The aim of this study was to determine the circulatory miRNA profile of variable stress-responding lambs (*n* = 112) categorized based on their cortisol levels as high (HSR, 336.2 ± 27.9 nmol/L), middle (MSR, 147.3 ±9.5 nmol/L), and low (LSR, 32.1 ± 10.4 nmol/L) stress responders post-LPS challenge (400 ng/kg *iv*). Blood was collected from the jugular vein at 0 (T0) and 4 h (T4) post-LPS challenge, and miRNAs were isolated from four animals from each group. An array of 84 miRNAs and 6 individual miRNAs were evaluated using qPCR. Among 90 miRNAs, there were 48 differentially expressed (DE) miRNAs (log fold change (FC) > 2 < log FC) in the HSR group, 46 in the MSR group, and 49 in the LSR group compared with T0 (control) samples. In the HSR group, three miRNAs, miR-485-5p, miR-1193-5p, and miR-3957-5p were significantly (*p* < 0.05) upregulated, while seven miRNAs, miR-376b-3p, miR-376c-3p, miR-411b-5p, miR-376a-3p, miR-376b-3p, miR-376c-3p, and miR-381-3p, were downregulated (*p* < 0.05) as compared to the LSR and MSR groups. Functional analysis of DE miRNAs revealed their roles in Ras and MAPK signaling, cytokine signaling, the adaptive immune system, and transcription pathways in the HSR phenotype, implicating a hyper-induced acute-phase response. In contrast, in the LSR group, enriched pathways included glucagon signaling metabolic regulation, the transportation of amino acids and ions, and the integration of energy metabolism. Taken together, these results indicate variation in the acute-phase response to an immune stress challenge, and these miRNAs are implicated in regulating responses within cortisol-based phenotypes.

## 1. Introduction

Stress is a physiological response induced by stressors, including microbial, social, physical, and environmental stimuli that activate various systems to restore homeostasis [1]. This stress response comprises a complex and integrated network of central neural and peripheral neuroendocrine responses to overcome induced stress [1]. The magnitude of the response varies among individuals and populations, which reflects genetic predisposition and experiences regulated by epigenetic factors. In terms of microbial stressors, research programs studying the stress response have presented mechanistic evidence of complex host–pathogen interactions leading to variability in the host response [2,3]. The recent increase in stress biology research within the livestock industry has recognized ‘stress’ as a major concern that must be addressed to mitigate negative outcomes on animal health, production, and welfare.

The hypothalamic–pituitary–adrenal (HPA) axis is marked as one of the key ‘stress’ axes that are activated during microbial exposure, and HPA activation culminates in the secretion of adrenal glucocorticoids (cortisol) as a physiologically relevant circulatory stress biomarker. Previously, variation in elevated glucocorticoid concentrations amongst individual animals and species was reported to result from genetic differences, prior experiences, and temperament [2]. Consistent individual differences in glucocorticoid levels imply differences in responsiveness to aversive situations, including the ability to cope with immunological challenges. Previous studies have reported considerable variation in HPA activation within populations, and the genetic contribution to this variable response was established by heritability estimates in several species, including sheep (h2 = 0.28) [4,5,6]. Thus, targeting this variability among individuals through genetic selection to mitigate the adverse effects of stress could be achieved by identifying and breeding resilient animals that can withstand existing and upcoming stressors.

Microbial stressors are the most common stressors for livestock species and have huge implications in terms of animal health and welfare. Among microbes, *Escherichia coli*-derived lipopolysaccharide (LPS) is a potent endotoxin that is commonly present in various livestock pathologies, such as mastitis, endometritis, and acidosis [7,8], and this pathogen imposes huge economic losses in the livestock industry. The stimulation of the HPA axis by exposure to LPS leads to the activation of the systemic acute-phase response, which is well studied [1,3,9,10,11]. Our previous ovine studies have characterized the LPS-induced acute stress response of lambs and demonstrated variability in individual stress responses [3,11,12]. LPS is a principal pathogen-associated molecular pattern (PAMP) primarily recognized by Toll-like receptor 4 (TLR4), which is expressed on mammalian leukocytes. The recognition of LPS by this pattern-recognition receptor triggers the innate inflammatory response, which ultimately leads to the activation of the HPA axis to help restore physiological homeostasis, in part by regulating the inflammatory response to make sure it is effective but not excessive and damaging to host tissues.

The interplay of small noncoding RNAs, especially microRNAs (miRNAs), in microbial- or environmental-induced stress has been highlighted in recent years, and insights into the modulation of both innate and adaptive immune responses by miRNAs have been gained [13,14,15,16]. MiRNAs are a class of small noncoding RNAs ranging from 20 to 22 nts that control post-transcriptional gene expression either by cleaving target mRNAs or by inhibiting the translation of specific proteins. MiRNAs regulate major biological processes, such as stress and immune responses, as well as reproduction. MiRNAs are found in biofluids, for example, blood, urine, plasma, and saliva [17,18]. Although pure RNA is prone to rapid degradation, miRNAs from biofluids show remarkable stability [19] due to being contained in exosomes. The process of miRNA secretion in exosomes is still largely unknown. Despite limited knowledge, it is well described that the miRNA patterns of biofluids change under pathological conditions [19,20,21]. Due to the sensitivity of detection and the possibility of multiplexing analyses for increased specificity, miRNAs offer an advantage as biomarkers. Lately, miRNAs have been used as diagnostic markers for several diseases, such as cancers and inflammatory and autoimmune diseases [22], and recently, we identified differentially expressed miRNAs in LPS-challenged lambs and their potentially targeted signaling pathways [11]. The present study aimed to determine the circulating miRNA profile associated with variable stress-responding lambs following acute LPS-induced systemic inflammation.

## 2. Materials and Methods

### 2.1. Endotoxin Challenge and Sample Collection

All experimental procedures were approved by the University of Guelph Animal Care Committee (AUP # 3436). A total of 112 healthy outbred Rideau-Dorset female lambs at 80-90 days of age were chosen and housed at the Ponsonby sheep research station, University of Guelph (Guelph, ON, Canada). The day before the experiment, randomly selected sheep were weighed and housed in individual pens with ad libitum access to hay and water. Sheep were intravenously (iv) challenged with 1 mL of a 400 ng/kg bolus dose of lipopolysaccharide (*Escherichia coli* O111: B4, Sigma, St. Louis, MO, USA). No animals showed signs of clinical disease at the beginning of the experiment, and the average body weight was 31.4 ± 5.6 kg (mean ± SD). Blood was collected from the jugular vein pre-challenge (T0) and 4 h post-challenge (T4) to measure serum cortisol and cytokine (IL-6 and IL-10) expression using ELISA [11]. The lambs were classified as high (HSR, ±1 SD from the mean, 336.2 ± 27.9 nmol/L), middle (MSR, ± 1 from the mean, 147.3 ±9.5 nmol/L), and low (LSR, ± 1 SD from the mean, 32.1 ± 10.4 nmol/L) stress responders based on their T4 cortisol concentration.

### 2.2. miRNA Isolation and cDNA Synthesis

To assess serum miRNA expression, a subset of animals (*n* = 4/group) from each of HSR, MSR, and LSR group were selected. MiRNAs were isolated from a total of 16 serum samples, 4 from each stress responder group at T4 post-challenge (4 × 3 = 12) and 4 randomly selected pre-challenge control samples at T0, using the miRNeasy Serum Advanced Kit (Qiagen, Hilden, Germany) following our previously described protocol, as described by Sharma et al. [11]. The diluted cDNA (1:11) was then subjected to a miRNA PCR array for amplification.

### 2.3. miRNA Expression Analysis

Seven candidate miRNAs were selected for qPCR expression studies since these miRNAs were previously associated with LPS-related disorders. The forward primer sequence (5′-3′) of miRNA 200b was GCTGACGGTGCTAATACTGCCT, and the primer sequences of the other six miRNAs (miR-145, miR-130, miR-223, miR-1246, miR-31, and miR-29b) were selected from our previous study [11]. The expression levels were determined with the 2^−ΔΔCT^ method [23] using CE-miR-39 spike-in as the normalizer on a real-time qPCR instrument (StepOne Plus, ABI, Lincoln, CA, USA).

In addition, a commercially available 384-well (4 × 96) ovine miScript-PCR-based array (Qiagen, Hilden, Germany) consisting of 6 miScript PCR controls (1-6 snoRNA/snRNA), a spike-in CE-miR-39 control, a reverse transcription control assay (miRTC), positive PCR controls (PPCs), and 84 ovine miRNA targets was used. The reaction mixture was prepared according to the manufacturer’s instructions and PCR-quantified on a ViiA 7 Real-Time PCR system (ABI, USA). The expression data analysis of the qPCR array was performed using the global mean procedure [24], as described in our previous study [11].

### 2.4. Target Gene Prediction and Pathway Analysis 

The identification of miRNA-targeted genes was performed using approaches described previously [11]; TargetScan (http://www.targetscan.org; accessed on 10 April 2021), mirDB (http://www.mirdb.org/index.html; accessed on 10 April 2021), and Diana tools (http://diana.imis.athena-innovation.gr/DianaTools/index.php?r=site/index; accessed on 10 April 2021) were used with selection criteria of cumulative weighted value < −0.4 for Targetscan and a target score >70 for miRDb. The commonly identified target genes were subjected to gene ontology (GO) annotation and enrichment analysis from three ontologies (molecular function, cellular component and biological process), which were carried out using WebGestalt (http://www.webgestalt.org/#; accessed on 10 April 2021, version 2019), and pathway analysis was performed using Panther, where *p* < 0.05 was used as the threshold criteria.

### 2.5. Statistical Analysis

To compare the expression levels of candidate miRNAs and the serum levels of cortisol, IL-6, and IL-10 in the T0 and T4 groups, the values were analyzed using a two-way ANOVA test followed by Tukey’s multiple comparison test and a mixed-effect model (REML), respectively, using GraphPad Prism Software 9.3.1. All data are presented as the mean ± standard error of the mean (SEM), and significance was determined at a *p*-value less than 0.05.

## 3. Results

**Figure 1:**
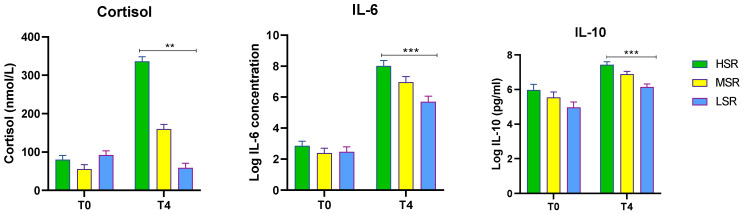
Basal (T0) and 4 h (T4) concentrations of serum cortisol, IL-6, and IL-10 in sheep before and after LPS (400 ng/kg) *i.v*. administration. Animals were classified as high (HSR), middle (MSR), or low (LSR) based on their T4 cortisol concentrations. Values are presented as means ± SEM. Significant differences are shown by asterisks (** *p* ≤ 0.01 and *** *p* ≤ 0.001) compared with the T0 (basal) levels.

The T0 and T4 LPS-induced serum cortisol, IL-6, and IL-10 concentrations for the variable stress-responding sheep are shown in Figure 1. There were no differences between the stress response groups in terms of the basal levels (T0) of cortisol, IL-6, and IL-10 (*p* > 0.05), but all were significantly induced at 4 h post-LPS challenge (*p* < 0.05).

**Figure 2:**
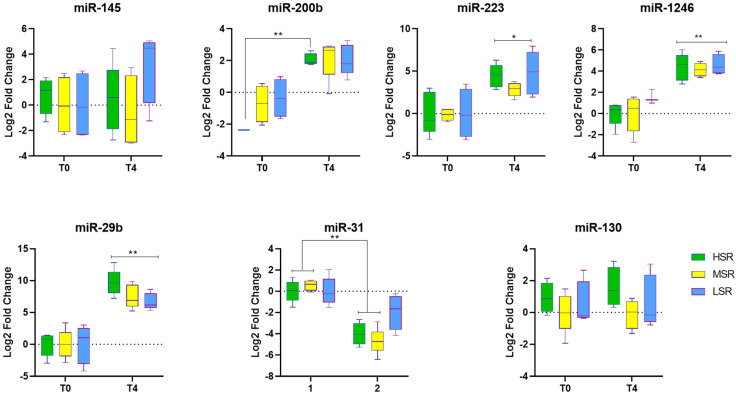
Fold change in expression of candidate ovine microRNAs (miR-145, miR-200b, miR-233, miR-1246, miR-29b, miR-31, and miR-130) before (T0) and 4 h (T4) after LPS (400 ng/kg) i.v. administration. The asterisks (* *p* ≤ 0.05 and ** *p* ≤ 0.01) indicate significant differences between T0 and T4.

**Figure 3:**
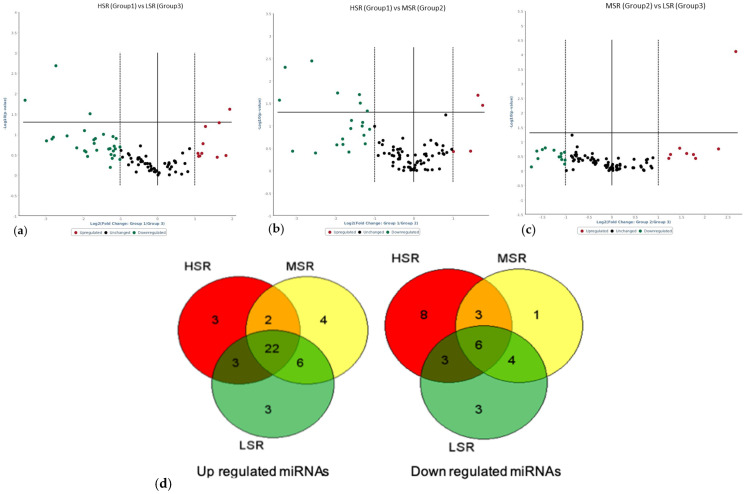
Volcano plots of differentially expressed miRNAs 4 h post-LPS (400 ng/kg) i.v. administration from variable stress-responding groups of sheep: (**a**) HSR vs. LSR, (**b**) HSR vs. MSR, and (**c**) MSR vs. LSR. (**d**) Venn diagram of genes commonly upregulated and downregulated in all groups. Number digits are numbers of miRNAs that are up/down regulated.

Comparisons were made between T4 and T0 (pre-challenge) samples to recognize miRNAs associated with the LPS immune challenge. The expression analysis of individual candidate miRNAs by qPCR showed that miR-29b, miR-1246, miR-223, and miR-200b were significantly induced (*p* < 0.05) by LPS in all stress-responding groups (Figure 2); however, no LPS-induced changes were observed for miR-145 and miR-130. For the miRNA arrays, the number of differentially expressed (2-fold change) miRNAs was 48 in the HSR group, 46 in the MSR group, and 49 in the LSR group. The number of upregulated (>2-fold change) miRNAs was 29 in the HSR group, 33 in the MSR group, and 34 in the LSR group; among these, 19 miRNAs were commonly induced (>2-fold change) across all stress-responding groups (Figure 3a–c; Table 1). Among the upregulated ovine miRNAs, three miRNAs (miR-485-3p, miR-543-3p, and miR-655-3p) were significantly expressed in all stress groups: HSR (+24.89-, +22.7-, and +6.60-fold, respectively), MSR (+7.36-, +23.77-, and +8.48-fold, respectively), and LSR (+6.51-, +22.09-, and +14.23-fold, respectively). However, only one ovine miRNA (miR-665-5p) was commonly reduced in all groups (*p* < 0.05), namely, HSR (-2.01-fold), MSR (-3.54-fold), and LSR (-2.07-fold) (Table 1).

To compare the miRNA expression among the variable stress-responding groups at T4 and identify the DE miRNAs specifically associated with each group, a comparative analysis was conducted for each group. On comparing HSR with other groups, three ovine miRNAs (miR-485-5p (+3.82-fold), miR-1193-5p (+2.43-fold), and miR-3957-5p (+3.14-fold)) were significantly (*p* < 0.05) upregulated in HSR animals. Conversely, three ovine miRNAs (miR-376b-3p (-6.6-fold), miR-376c-3p (-3.5-fold), and miR-411b-5p (-11.69-fold)) were downregulated (*p* < 0.05) in the HSR group as compared to LSR. Comparisons between HSR and MSR revealed that four miRNAs (miR-376a-3p (-2.28-fold), miR-376b-3p (-6.08-fold), miR-376c-3p (-2.62-fold), and miR-381-3p (-3.85-fold)) were downregulated (Table 2), whereas, when comparing MSR with LSR, just one ovine miRNA (miR-758-3p, +6.37-fold) was significantly (*p* < 0.05) induced.

**Figure 4:**
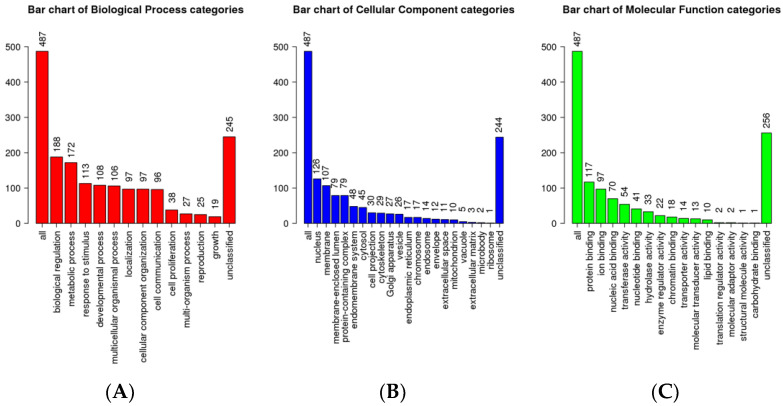
GO analysis of DE miR target genes. (**A**) Biological processes for differential miRNAs. (**B**) cellular components for differential miRNAs. (**C**) Molecular functions for differential miRNAs.

Subsequently, the target genes of upregulated ovine miRNAs (miR-485-5p, miR-1193-5p, miR-3957-5p, and miR-758-3p) in each group were identified using TargetScan and Diana tools and subjected to GO analysis using Web-GSTAL (Figure 4). Further pathway analysis was performed using the Panther database. The top pathways targeted by upregulated miRNAs in the HSR group were Ras and MAPK signaling, cytokine signaling, adaptive immune system, and transcription pathways (Table 3), whereas, in the MSR group, the transport of small molecules and N-acetylglucosamine metabolism were significantly highlighted. In contrast, in the LSR group, enriched pathways included glucagon signaling in metabolic regulation, the transportation of amino acids and ions, and the integration of energy metabolism (Table 3).

## 4. Discussion

Variation in stress responsiveness is determined by genetics and epigenetic changes and is associated with variation in the immune response. For example, Naylor et al. demonstrated different cytokine levels and leukocyte numbers in variable stress-responding sheep following an LPS immune challenge [12]. These variable responses to an LPS immune challenge could present an effective strategy to increase the stress resiliency of sheep through genetic selection.

As a potent agonist of TLR4, LPS inflammation can activate TLR4 signaling pathways and induces the expression of the inflammatory cytokines TNF-α, IL-1, and IL-6 [3]. Cortisol is one of the most reliable stress markers, and biologically, it causes a variety of physiological and cognitive changes crucial for stress regulation. Ranking animals as extreme groups based on cortisol levels could help us to better understand individual variations in induced responses, which could further be explored in terms of enhanced resilience. The present investigation also identified circulating miRNAs that were differentially expressed in variable cortisol-responding sheep following the LPS challenge, which could in part contribute to variations in the acute-phase response. These circulatory biomarkers possess positive attributes in that they are readily measurable, obtained non-invasively, highly stable, and inducible under compromising states [25]. Emerging research indicates that stress conditions can alter the biogenesis of miRNAs, the expression of mRNA targets, and the activities of miRNA–protein complexes [26]; thus, the present variable responses could result from the altered expression of these complexes. Lately, miRNAs have been considered new biomarkers in diseases [27], which implies that miRNAs could alter the inflammatory response.

**Figure 5:**
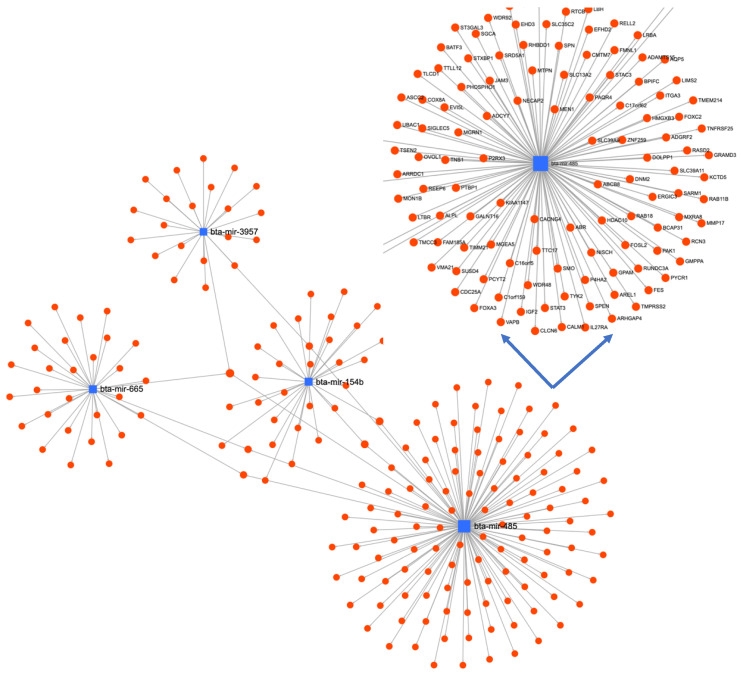
miRNA-mRNA-miRNA interaction network of differentially expressed miRNAs using MirNet online tool.

We have identified three ovine miRNAs (miR-485-3p, miR-1193, and miR-3957) that were specifically upregulated and only one (miR-376b-3p) that was downregulated in the HSR group compared to other groups. Of these, miR-485 and miR-3957 were present in a common miRNA network (Figure 5), regulating several genes and inducing immune-related pathways, including MAP kinase and cytokine signaling pathways, which is further supported by high cytokine levels in the HSR group. These miRNAs were also significantly differentially expressed in our previous investigation in LPS-treated lambs [11]. The expression of oar-miR-485-3p was significantly higher in the HSR group compared to MSR and LSR. The overexpression of miR-485-3p has been reported to induce T-cell activation, with a significant increase in activated CD8+ T cells, and to upregulate NF-κB and PI3Kδ gene expression, promoting the production of pro-inflammatory cytokines [28,29]. Additionally, its overexpression modulated the Th1/Th2 response by inducing differentiation toward a Th2 phenotype, which is supported by the determined cytokine levels in the present study.

In the MSR group, only one miRNA, miR-758-3p, was upregulated when compared to the LSR group, whereas three miRNAs (miR-376b-3p, miR-376c-3p, and miR-411) were upregulated in LSR in comparison to the HSR group. The crosstalk between lipid metabolism and innate immunity pathways has a significant role in the development or prevention of atherosclerosis, as suggested previously [30]. The specific upregulation of miR-758-3p in the MSR group could be associated with a moderate increase in cortisol and inflammatory cytokine levels; however, its expression remained unchanged in the HSR group, which had high cortisol and cytokine levels. Therefore, it would be imperative to further study its role in stress resiliency among variable stress responders.

On the other hand, miR-411a-3p/5p were significantly upregulated in the low-cortisol LSR group, which has recently been implicated in the inhibition of inflammation and enhanced recovery in rats following an LPS challenge [31]. This study reported that miRNA-411 attenuated NFκB, thus improving inflammation via JNK pathway inhibition by negatively targeting the pro-inflammatory cytokine IL-18 [31]. Our previous study showed lower cortisol and pro-inflammatory cytokine levels in the LSR group [12], which aligns well the with high expression of miR-411.

The other miRNAs (miR-1246, miR-200, miR-223, and miR-29b) were differentially expressed in all variable groups post-LPS immune challenge. These miRNAs were reported earlier in sepsis patients and in many studies aiming to understand the regulation of signaling molecules in the TLR-4 pathway upon LPS exposure [32,33]. These miRNAs were previously associated with immunity and cellular functioning in LPS-stimulated bovine monocytes [34,35], and they target different signaling molecules in the TLR4 pathway that further modulate the efficiency of TLR4 signaling and affect the host innate defense against microbial pathogenesis [11,36]. We found that the expression of miRNA-1246 was significantly higher in the LSR group compared to HSR post-LPS immune challenge. Recently, miR-1246 was reported to regulate cellular apoptosis in dairy cattle under heat stress and is suggested to have a potential role in stress management [37]. On the other hand, miRNA-29b was higher in HSR animals compared to the LSR group, and it is known to regulate glucose metabolism and natural killer cell function and is involved in the inhibition of the Th1-cell-mediated immune response [38,39]. These results agree well with the high levels of anti-inflammatory cytokines (IL-10 and IL-4) in the HSR group [12]. Moreover, the high abundance of miRNA-29 expression was previously associated with high-performance dairy cows and is known to increase the availability of branched-chain essential amino acids, hence activating the mTOR signaling pathway [40]. Given this, it would be interesting to investigate the performance of these variable stress responders in terms of meat production, milk production and quality, and neonatal health.

In conclusion, the present study identified several key miRNAs associated with the acute-phase response of lambs post-LPS challenge. There were 48 differentially expressed miRNAs in the HSR group, 46 in the MSR group, and 49 in the LSR sheep group as compared to the control group (T0). The extreme phenotypes, HSR and LSR, had different expression patterns of miRNAs, and functional analysis depicted different classes of pathways enriched in these phenotypes. In the HSR group, three miRNAs (miR-485-5p, miR-1193-5p, and miR-3957-5p) were significantly (*p* < 0.05) upregulated, while three miRNAs (miR-376b-3p, miR-376c-3p, and miR-411b-5p) were downregulated (*p* < 0.05) as compared to the LSR group. Together, our study indicates that miRNA expression plays an indispensable role in the stress response. The identified miRNAs play an important role in modulating immune and stress responses and could contribute to individual differences in stress resiliency. Yet, in many of these cases, the molecular mechanisms remain unclear and should be investigated further to elucidate these fundamental roles of miRNAs in controlling mRNA regulation during LPS stress.

## Figures and Tables

**Table 1:** Differentially expressed (T4 versus T0) ovine serum miRNAs post-LPS immune challenge for HSR, MSR, and LSR sheep. Values represent fold change (FC) followed by the corresponding *p*-value.

S.No		HSR Log FC	MSR Log FC	LSR Log FC
Upregulated miRNAs				
1	oar-miR-485-3p	**24.894** **(0.009)**	**7.367** **(0.034)**	**6.518** **(0.0070)**
2	oar-miR-543-3p	**22.743** **(0.028)**	**23.775** **(0.007)**	**22.089** **(0.022)**
3	oar-miR-655-3p	**6.601** **(0.033)**	**8.480** **(0.0009)**	**14.236** **(0.020)**
4	oar-miR-3957-5p	**4.631** **(0.015)**	5.156 (0.359)	1.4751 (0.344)
5	oar-miR-329b-3p	2.402 (0.051)	1.777 (0.169)	5.456 (0.172)
6	oar-miR-369-3p	13.139 (0.393)	**32.476** **(0.047)**	**35.838** **(0.05)**
7	oar-miR-411a-5p	4.871 (0.087)	**9.739** **(0.026)**	7.021 (0.158)
8	oar-miR-411a-3p	4.504 (0.110)	**6.420** **(0.023)**	10.881 (0.132)
9	oar-miR-487b-3p	5.588 (0.098)	**7.206** **(0.023)**	9.443 (0.148)
10	oar-miR-758-3p	1.107 (0.630)	**3.332** **(0.001)**	−1.912 (0.092)
11	oar-miR-668-3p	8.781 (0.08)	**5.237** **(0.05)**	**4.847** **(0.05)**
12	oar-miR-376c-3p	1.329 (0.90)	3.475 (0.090	**4.650** **(0.05)**
13	oar-miR-381-3p	−1.668 (0.466)	**2.308** **(0.05)**	−1.041 (0.810)
Downregulated miRNAs				
14	oar-miR-665-5p	**−2.015** **(0.014)**	**−3.547** **(0.003**	**−2.074** **(0.05)**
15	oar-miR-379-5p	**−2.034** **(0.039)**	−1.603 (0.121)	−1.480 (0.147)
16	oar-miR-154b-5p	**−1.966** **(0.057)**	**−1.964** **(0.050**	−1.837 (0.097)
17	oar-miR-3958-5p	1.045 (0.524)	**−2.612** **(0.0660**	**−2.896** **(0.068)**
18	oar-miR-496-5p	−1.636 (0.23)	**−2.122** **(0.05)**	**−2.030** **(0.0310**
19	oar-miR-329b-5p	−1.180 (0.993)	1.003 (0.808)	**−2.745** **(0.050)**

**Table 2:** Differentially expressed ovine miRNAs within variable stress-responding groups. Values represent fold change (FC), followed by the corresponding p-value.

	HSR vs. LSR	HSR vs. MSR	MSR vs. LSR
Upregulated	oar-miR-485-3p (3.82, 0.0243) oar-miR-1193-5p (2.43, 0.064) oar-miR-3957-5p (3.14, 0.052)	oar-miR-485-3p (3.38, 0.0352) oar-miR-1193-5p (3.11, 0.021)	oar-miR-758-3p (6.37, 0.0000794)
Downregulated	oar-miR-376b-3p (−6.6, 0.0020) oar-miR-376c-3p (−3.5, 0.0313) oar-miR-411b-5p (−11.69, 0.014)	oar-miR-376a-3p (−2.28, 0.047) oar-miR-376b-3p (−6.08, 0.003) oar-miR-381-3p (−3.85, 0.018) oar-miR-758-3p (−3.01, 0.076)	

**Table 3:** List of top enriched LPS-induced pathways identified in lambs (treated vs. control) and within HSR and LSR groups.

S. No	Term	Corrected *p*-Value (FDR)	Database
*Pathways enriched in all treated groups compared to control*			
1	MAPK signaling pathway	0.00000000775	KEGG PATHWAY
2	Signaling pathways regulating pluripotency of stem cells	0.0000364	KEGG PATHWAY
3	EGFR tyrosine kinase inhibitor resistance	0.0000542	KEGG PATHWAY
4	PI3K-Akt signaling pathway	0.0000674	KEGG PATHWAY
5	Longevity regulating pathway	0.00012941	KEGG PATHWAY
6	Ras signaling pathway	0.00014182	KEGG PATHWAY
7	Longevity regulating pathway—multiple species	0.0003686	KEGG PATHWAY
8	Autophagy—animal	0.00051481	KEGG PATHWAY
9	AMPK signaling pathway	0.00120604	KEGG PATHWAY
10	mTOR signaling pathway	0.00154106	KEGG PATHWAY
*Enriched pathways specific to HSR group*			
1	Gene expression (transcription)	0.00256736	Reactome
2	Immune system	0.00740769	Reactome
3	Ras signaling pathway	0.0105625	KEGG PATHWAY
4	Signal transduction	0.02158305	Reactome
5	MAPK signaling pathway	0.02705895	KEGG PATHWAY
6	Adaptive immune system	0.03477747	Reactome
7	Cytokine signaling in immune system	0.00232352	Reactome
*Enriched pathways specific to LSR group*			
8	Metabolic pathways	0.01151519	KEGG PATHWAY
9	Glucagon signaling in metabolic regulation	0.04716364	Reactome
10	Transport of inorganic cations/anions and amino acids/oligopeptides	0.06204974	Reactome
11	Integration of energy metabolism	0.06204974	Reactome
12	Post-translational protein modification	0.07675048	Reactome
13	Longevity regulating pathway	0.07740826	Reactome

## Data Availability

The datasets used and analyzed during the current study available from the corresponding author upon request.
